# How are patients with rare diseases and their carers in the UK impacted by the way care is coordinated? An exploratory qualitative interview study

**DOI:** 10.1186/s13023-020-01664-6

**Published:** 2021-02-10

**Authors:** Amy Simpson, Lara Bloom, Naomi J. Fulop, Emma Hudson, Kerry Leeson-Beevers, Stephen Morris, Angus I. G. Ramsay, Alastair G. Sutcliffe, Holly Walton, Amy Hunter

**Affiliations:** 1 Genetic Alliance UK, Third Floor, 86-90 Paul Street, London, EC2A 4NE UK; 2 The Ehlers-Danlos Society, Office 7, 35-37 Ludgate Hill, London, EC4M 7JN UK; 3 Department of Applied Health Research, University College London, London, UK; 4 Department of Public Health and Primary Care, Institute of Public Health, University of Cambridge, Forvie Site, Robinson Way, Cambridge, UK; 5 Alstrom Syndrome UK, 4 St Kitts Close, Torquay, TQ2 7GD Devon UK; 6 UCL and Great Ormond Street Institute of Child Health, London, UK

**Keywords:** Care coordination, Financial impact, Psychosocial impact, Undiagnosed conditions, Patient experience, Carer experience, Care coordinators, Multi-disciplinary clinics, Specialist care, Care plans

## Abstract

**Background:**

Care coordination is considered important for patients with rare conditions, yet research addressing the impact of care coordination is limited. This study aimed to explore how care coordination (or lack of) impacts on patients and carers. Semi-structured interviews were conducted with 15 patients and carers/parents in the UK, representing a range of rare conditions (including undiagnosed conditions). Transcripts were analysed thematically in an iterative process.

**Results:**

Participants described a range of experiences and views in relation to care coordination. Reports of uncoordinated care emerged: appointments were uncoordinated, communication between key stakeholders was ineffective, patients and carers were required to coordinate their own care, and care was not coordinated to meet the changing needs of patients in different scenarios. As a result, participants experienced an additional burden and barriers/delays to accessing care. The impacts described by patients and carers, either attributed to or exacerbated by uncoordinated care, included: impact on physical health (including fatigue), financial impact (including loss of earnings and travel costs), and psychosocial impact (including disruption to school, work and emotional burden). Overall data highlight the importance of flexible care, which meets individual needs throughout patients’/carers’ journeys. Specifically, study participants suggested that the impacts may be addressed by: having support from a professional to coordinate care, changing the approach of clinics and appointments (where they take place, which professionals/services are available and how they are scheduled), and improving communication through the use of technology, care plans, accessible points of contact and multi-disciplinary team working.

**Conclusion:**

This study provides further evidence of impacts of uncoordinated care; these may be complex and influenced by a number of factors. Approaches to coordination which improve access to care and lessen the time and burden placed on patients and carers may be particularly beneficial. Findings should influence future service developments (and the evaluation of such developments). This will be achieved, in the first instance, by informing the CONCORD Study in the UK.

## Background

Following a recent review of definitions [1], care coordination for chronic and rare conditions has been defined as follows: [p. 8]

*Coordination of care involves working together across multiple components and processes of care to enable everyone involved in a patient’s care (including a team of healthcare professionals, the patient and/or carer and their family) to avoid duplication and achieve shared outcomes, throughout a person’s whole life, across all parts of the health and care system. Coordination of care should be family-centred, holistic (including a patient’s medical, psychosocial, educational and vocational needs), evidence-based, with equal access to coordinated care irrespective of diagnosis, patient circumstances and geographical location.*

In the United Kingdom (UK), coordinated care for patients with rare conditions is provided in different ways such as through specialist centres, care coordinators, multi-disciplinary teams, care plans and ‘one-stop-shops’/residential clinics where patients can access a range of services during one hospital visit [2]. However, services are not delivered in the same way across the country and experiences can depend on the condition, patients’ age, and their location [2–4] and many rare disease patients are not aware of or do not access a specialist centre [4].

Improving the coordination of care for rare conditions is a priority for UK government strategy [5] and NHS (National Health Service) England state in their implementation plan [6] that specialised services should include: a person responsible for coordination, an alert card and an active transition from paediatric to adult services. However, progress is limited [2, 7] and more research is needed in the UK, and across Europe, to demonstrate the effectiveness of specific elements of rare disease plans on patient outcomes [8].

Research has indicated that coordinating care for rare conditions may be more complex than it is for common chronic conditions, for example, coordination may be required in the context of no diagnosis and/or a lack of awareness and expertise in the condition [1]. An individual’s health needs are often shared over clinical subspecialties [9] and they are also likely to require coordination across a range of other support needs and professionals (including social care and education) [1].

Limited research suggests that there may be both financial and non-financial ‘hidden’ impacts for patients and their families, associated with how care is coordinated [2, 3]. A range of challenges related to how care is managed have been identified including:

(a) psychological and emotional challenges resulting from high turnover of healthcare professionals and a lack of information and knowledge amongst professionals [10],

(b) stress and financial concerns for parents due to the burden associated with planning and coordinating care to meet the unique needs of their children [11] and,

(c) a substantial time burden for patients and carers, in part, because of coordinating their care [12].

Specialised treatment centres/teams have been perceived to have positive outcomes, including being characterised with more experience and knowledge, yielding better patient satisfaction [10] and specialist multi-disciplinary ‘one stop shops’ have demonstrated more organised, personalised and holistic care, resulting in better treatment compliance [9]. However, a number of challenges in assessing models of care coordination for rare diseases have been identified by economists including small numbers of patients, making it challenging to know how to organise care appropriately [13].

So, while care coordination is considered important for patients with rare conditions, there is a lack of research which addresses care coordination in the context of rare conditions [1]. More specifically, there is a dearth of evidence on the impact of different approaches to care coordination for such patient groups [14] including the impact on patients and their families [2, 3]. It has also been noted that where research does exist, it tends to be condition specific, and there is a lack of qualitative research which addresses the shared experience of those with rare conditions [10].

Given this paucity of data in this area, the aim of this study was to (i) explore how rare disease patients and their carers are impacted by how their care is, or is not, coordinated, and (ii) explore the factors which might influence effective care coordination from the perspective of patients and carers. Qualitative methodology is ideally suited for investigating psychological, emotional and social impacts of living with a rare condition [10]. Using qualitative research (with patients and carers affected by a range of conditions) to understand what the impacts of care coordination might be, is an important initial step in the future evaluation and design of different models of care for patients with rare conditions.

## Methods

### Ethical approval

The research was approved by University College London’s research ethics committee (project ID 8423/002).

### Design

This was an exploratory qualitative interview study of patients affected by rare conditions (including undiagnosed conditions) and their carers. Data was analysed thematically [15], in an iterative process, using both inductive and deductive approaches.

### Recruitment

We recruited patients and carers (including parents of patients and spouses/partners of adult patients) affected by rare conditions in the UK. Participants were recruited from charity networks (Genetic Alliance UK, Rare Disease UK and Syndromes Without A Name (SWAN) UK) using a purposive sampling method. An advert, inviting interested individuals to contact the research team, was disseminated via email (newsletters and members’ updates), social media, and charity websites.

Purposive sampling methods are widely used in qualitative research so that the most ‘information-rich’ cases can be identified and selected [16]. The purpose of our analysis was to get a rich understanding of the experience of a number of patients and carers—*not* to be representative of all people with an experience of rare diseases. However, the approach was adopted to gather data on impacts from several different perspectives including from those experiencing different approaches to coordination. In October 2018, participants were selected from 60 individuals. A sample was specifically chosen to include: patients and carers, those with and without a diagnosis, and those with a range of coordination experiences (including those who had a professional coordinating their care and those who coordinated care themselves, those who attended a specialist centre and those who did not). Participants were also selected to represent a range of ages and locations across the UK. Further details of participants’ characteristics are included in the results section.

### Data collection

Interviews were semi-structured and conducted by telephone or Skype. All participants received a participant information sheet and consent form via email, and were given the opportunity to discuss the study and ask the researcher questions, before they agreed to take part. Interviews were scheduled at a time convenient for the participant. Verbal informed consent was taken and recorded at the start of the interviews. Fifteen interviews were conducted (by AS) between October 2018 and January 2019 (telephone: n = 14, Skype: n = 1). Interviews ranged from 34 to 93 min (mean: 51 min).

The interview questions were informed by findings of a feasibility study exploring the hidden costs of rare diseases [2] and included questions about how care was currently organised and how individuals would like it to be organised, what was important to them in relation to care coordination (and how this might change over time) and the costs and benefits associated with how care is coordinated (see Additional file 1).

Interviews were recorded using an encrypted Dictaphone and transcribed verbatim by a professional transcribing company. Transcripts were read and checked for accuracy (by AS). Personal information (such as names and places) were removed prior to analysis.

### Analysis

The primary researcher (AS) read transcripts and made initial notes. Codes were identified from a previous feasibility study (e.g. different types of costs and benefits such as ‘financial’ and ‘psychosocial’) [2]. In addition, two members of the research team (AS and EH) each open-coded two transcripts to identify new codes. A draft coding framework, including both anticipated and emergent codes, was developed. Three transcripts were then independently coded by two researchers (AS and HW), who met to share their coding. Any disagreements were discussed until a consensus was met. The revised coding frame was then applied to all remaining transcripts by the primary researcher (AS) using QSR NVivo 12 [17] qualitative data analysis programme. To develop themes, a process of Iterative Categorisation (IC) [18] was followed to systematically reduce, review and summarise the data. Ideas were shared with the study team (several members of which were experts from the clinical and voluntary sector).

## Results

Participant characteristics were collected prior to interview. Participants included both patients affected by rare diseases (7) and carers (8).^1^ Carers were all informal carers (they were not carers by profession—they were either the parent of a child with a rare disease or the spouse/partner of an adult with a rare disease). Participants were a range of ages: one participant was aged between 18 and 25, 12 participants were aged between 26 and 59, and two were aged 60 or over. Patient age (age of patients cared for) also ranged: six were under 18 and two were aged between 26 and 59. Participants lived in different regions across the UK including East Midlands (3), East of England (2), Greater London (2), North East (1), North West (1), South East (3), South West (2) and Scotland (1). Further characteristics, including whether the patient had a diagnosis, who coordinates care and whether participants had access to a specialist centre,^2^ are shown in Tables 1 and 2.

**Table 1:** Participants’ characteristics

	Patients	Carers		Total
		Parents of children	Partner/spouse of adult patient	
Diagnosed	5	4	2	11
Undiagnosed	2	2	0	4
Total	7	6	2	15

**Table 2:** Access to a specialist centre/who coordinates care

	Who coordinates the patient’s care?			Total
	Patient/carer coordinates care	Patient/carer coordinates care with professional	Professional/service coordinates care	
Does the patient access a specialist centre?				
Yes	3	0	4	7
No	4	2	0	6
Don’t know	2	0	0	2
Total	9	2	4	15

Findings to address the aims are grouped under three main headings: Experiences of uncoordinated care for patients with rare conditions, How uncoordinated care impacts on patients and carers, and Examples of coordinated care and approaches to reduce the negative impacts of imperfect care coordination on patients and carers.

### Experiences of uncoordinated care for patients with rare conditions

**Figure 1:**
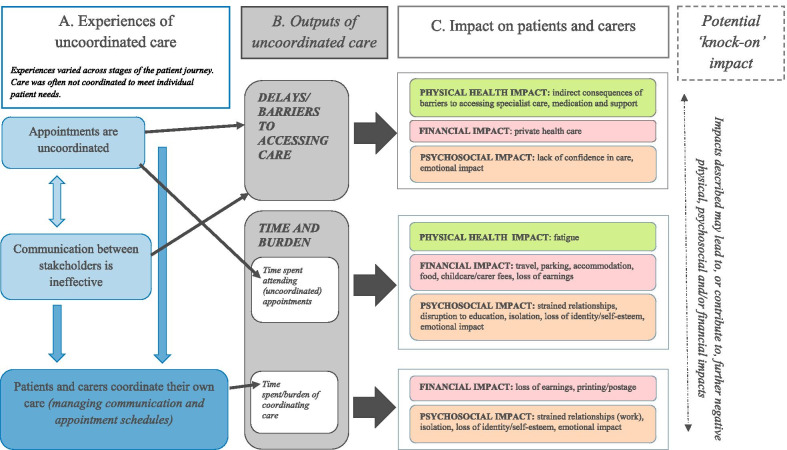
Patients’ and Carers’ experiences of uncoordinated care and their impact

Participants described a range of experiences of uncoordinated care (see Fig. 1 ‘A. Experiences of uncoordinated care’). These included:

- Uncoordinated appointments: frequent appointments to see different professionals/services across different NHS settings, some of which were located far away from home (i.e. at specialist centres), lack of choice about when or where their appointments took place, and appointments with one professional at a time (with little evidence of medical and non-medical services being offered in the same clinic).
- Ineffective communication between stakeholders: lack of communication/team approach across various care professionals (particularly between those in specialist centres and local teams), no point of contact to approach with queries, problems relating to information sharing (particularly the timeliness of information sharing), and limited use of care plans.
- Patients and carers coordinating their own care: patients and carers undertaking a number of tasks including chasing services, holding information and facilitating information sharing.  Many reported being the main coordinator of care.

In addition, the data showed that care is not coordinated to meet the needs of different patients and scenarios. Care was uncoordinated at particular stages of the patient journey—in particular, there were challenges associated with: emergencies or acute episodes (with a lack of awareness locally and difficulties accessing timely treatment), pre and post diagnosis (particularly in establishing care and support), following discharge from hospital (and receiving the appropriate care within the community), and transitioning from paediatric to adult services. Similarly, care was considered uncoordinated if it did not meet individual patient needs (for example, appointments were not scheduled to allow for minimum disruption to work/school). Experiences of uncoordinated care therefore varied across individuals and stages of the patient journey.

### How uncoordinated care impacts on patients and carers

Uncoordinated care resulted in both delays/barriers to accessing care and a burden on patients and carers (see Fig. 1 ‘B. Outputs of uncoordinated care’), which in turn had several negative impacts for patients and carers (see Fig. 1 ‘C. Impacts on patients and carers’).

#### How did uncoordinated care influence patients’ access to care?

Interviewees reported that uncoordinated appointments and ineffective communication between stakeholders impacted on their ability to access care and access that care in a timely way. ‘Care’ included care that is specialist (care from professionals/settings which specialise in the condition and have specialist knowledge), care in different settings (including in local settings), non-medical care and support, proactive care, accurate diagnosis and treatment/medications.

Seeing numerous professionals over several different appointments resulted in delays in decision making about their care. Delays were also evident as a result of ineffective communication between professionals and information sharing across different trusts/services—interviewees reported wasting time during appointments updating professionals and/or waiting for them to chase results.

Initiatives which facilitated communication between professionals (e.g. care plans or multi-disciplinary meetings) were limited. Therefore, even for those patients who accessed specialist care (i.e. via a specialist centre), the location and accessibility of specialist care/advice combined with the lack of effective communication between local and specialist teams resulted in challenges, particularly in accessing local care during acute scenarios.

*I would imagine a lot of people with rare diseases find this: that when they turn up at their local hospital whoever is on-shift generally has no idea what you’re talking about so you always have to go back to your Consultant [and] there could be delays [carer, diagnosed]*

More than one interviewee reported facing delays in accessing their medication, again, as a result of ineffective communication between specialists and local services. Patients and carers reported that health and other sectors, such as social care, did not communicate with each other, sometimes preventing vital access to non-medical support.

*it’s terrible the coordination between the Social Work side of things and the Health side of things. It took us 18 months actually to get a Social Worker, which seems crazy given that my son has a really profound disability. [carer, diagnosed]*

#### How did the challenges associated with access to care impact negatively on patients and carers?

Such barriers and delays are likely to have consequences for patients and carers. First, it can have a negative impact on a patient’s physical health, particularly if diagnosis or treatment is delayed. Second, it can have a financial impact on families—participants reported paying for private health care as a last resort and as a result of delays or fighting for access to care. Third, it can have psychosocial impacts –patients and carers reported on the emotional impact of having to fight for their care and they experienced a loss of confidence in the care they received.

*our main problem was obviously getting access to a doctor… who could do the appropriate tests… when [respondent’s son1] developed that squint… which was kind of about a year before, he should have been referred to a neurologist at that point. [carer, diagnosed]*

It is important to note that impact on physical health is also likely to have further ‘knock on’ impacts for patients and carers. For example, poorer physical health could result in the need for more medical intervention (which, if uncoordinated could magnify many of the issues already faced) and increased challenges associated with daily activities such as going to work (carrying further financial and psychosocial costs).

#### How did uncoordinated care create additional burden on patients and carers?

Patients and carers described the time and burden placed upon them to attend frequent and uncoordinated appointments (patients and carers spent significant time travelling to and attending various appointments) and coordinate their own care (supporting communication/information exchange and organising the vast appointment schedule).

In the absence of care coordination (and tools such as coordinators or care plans) families described having to adopt a proactive approach themselves to ensure they received the right care. This involved spending significant amounts of time chasing services for results, appointments and advice. One parent described their care coordinating role as a full-time job, with no pay.

*I’m the one that chases appointments and makes sure that we’re where we’re supposed to be … a huge amount of work but how can that really be improved? [carer, diagnosed]*

Managing information relating to the patient's condition and their care was a major task for families. Reports suggested that the records kept by professionals were sometimes incomplete or inaccurate. As a result, patients and carers were often required to update or correct professionals at each appointment. Some kept detailed paper records at home, rather than relying on the records kept by professionals.

*…If I ever got hit by a bus, we'd be screwed. Well, I wouldn't be obviously, I'd be completely blissfully unaware, but he would be stuffed because all of this stuff is in my head. [carer, undiagnosed]*

The time and burden associated with attending appointments and managing a care schedule is likely to affect all patients to some extent (including those with positive experiences of coordinated care). However, interviewees suggested that the costs were increased by (a) the uncoordinated nature of appointments—they were required to travel far and frequently for services which could, in theory, be offered locally and/or in one visit rather than several and (b) a lack of effective communication between the various professionals involved across specialities and locations.

#### How did the additional burden impact negatively on patients and carers?

Participants reported that appointment schedules can have a negative impact on a patient’s physical health. This was a particular issue for those whose condition caused fatigue. In addition, participants reported financial costs associated with attending appointments, such as those for travel and parking, accommodation (if the distance was too great to complete a return journey on the same day), food and fees relating to childcare (e.g. for patients’ siblings whilst parents were attending appointments) and carers (e.g. professional carers required to support parents when travelling and attending appointments). Psychosocial costs of attending appointments were also reported—for example, young patients missed time at school as a result of attending appointments.

*Obviously, it’s got a financial cost, but there’s a physical cost there, you know, having to go to extra appointments when I needn’t have to. [patient, diagnosed]*

Patients and carers frequently referred to the impact of attending uncoordinated appointments and coordinating their care on work and employment. In part, this was a further financial cost to families (i.e. a loss of earnings from reducing their hours, changing the nature of their role at work, or leaving paid employment to cope with the demands). Disruption to work also carried a psychosocial cost. Participants talked of strained relationships with colleagues and managers whilst negotiating time off and reducing hours, not having a break because they were using annual leave exclusively for appointments, and a loss of identity and self-esteem as a result of giving up their job and ‘independence’. This again demonstrates the multidirectional nature of some of the impacts—there may be several ‘knock on’ impacts for patients and carers. In this instance, the financial impact of losing earnings had a psychosocial impact on participants.

*…it has sort of an impact on your self-esteem, doesn’t it, because prior to having children I was a high-flyer and I was very independent and I earned a lot of money… whereas all of that has gone now; we’re living off the savings that my husband earned, I’m totally dependent on that and totally dependent on him. [carer, diagnosed]*

The emotional impact of managing a rare condition, in particular taking on the role of care coordinator, was also discussed by patients and carers. Words used to describe how the workload and burden made them feel included: exhausted, strained, frustrated, worried, suicidal, and terrified. Parents, in particular, discussed feeling anxious about being the ‘expert’ and being responsible for looking out for symptom changes and receiving little support. Isolation was also a common theme amongst parents, which was exacerbated by their workload (e.g. they did not have the time to socialise).

*The way that it’s been coordinated has probably added to the stress… having to be that person that is chasing everything, that’s definitely added to the stress. [carer, diagnosed]*

*it’s absolutely relentless and exhausting and heart-breaking. I’m on my seventh ring binder upstairs with all of the letters from the diagnoses and the medicine sheets…I’m just worried if I forget something. [carer, undiagnosed]*

There were also additional administration costs for families of printing and posting paperwork (e.g. the costs of record keeping and sharing paperwork with relevant individuals and bodies).

Participants reported having to rely on others within their family for things such as support coordinating care and providing childcare for children whilst attending appointments. Therefore, the non-financial and psychosocial impacts were also felt by wider family members who may have to support families practically and emotionally.

### Examples of coordinated care and approaches to reduce the negative impacts of imperfect care coordination on patients and carers

The findings above suggest that how care is coordinated can have several consequences for patients and carers. Improving or changing the way in which care is coordinated could improve access to care *and* reduce the time/burden experienced by patients and carers. In turn, this would reduce the negative (physical, financial and psychosocial) impacts described above. The data below (summarised in Table 3) emerged from examples of good coordination (currently experienced by participants) and/or participants’ suggestions for how coordination could be improved.

**Table 3:** A summary of how care could be coordinated to reduce the negative impacts felt by patients and carers

How care is/could be coordinated	What that might entail	How the approach might affect access to care and/or burden on patients and carers	Illustrative quotes from examples of coordinated care
Patients and carers have the support of a professional coordinator	Facilitating the communication and information exchange between key stakeholders	Improves access to care (e.g. helping exchange of information between specialist and local providers) Reduces time/burden on patients/carers associated with coordinating care and attending uncoordinated appointments	*I think without me having my [condition specific] specialist nurse coordinating the care… really taking some of the weight off, and doing a lot of the bread and butter… making sure I’m where I’m meant to be at the right time, and that the right doctor has got the right information… mum doesn’t have to deal with [that]…and I’m thankful. [patient, diagnosed]*
	Scheduling appointments in a convenient way, to meet patient needs	Reduces time/burden on patients/carers associated with coordinating care and attending uncoordinated appointments	
	Acting as a point of contact, including between appointments	Improves access to care (e.g. supporting patients to access specialist advice in between appointments)	
	Facilitating and providing additional support when required in patient journey	Improves access to care (e.g. signposting to relevant charities)	
The organisation of appointments meets the needs of patients and carers	Locally and remotely where possible	Reduces time/burden on patients/carers associated with attending uncoordinated appointments	*…there are instances where it has been coordinated well by being able to condense all my appointments into one day, which is obviously much kinder on the bank balance… Obviously, it’s got a financial cost, but there’s a physical cost there, you know, having to go to extra appointments when I needn’t have to. [patient, diagnosed]*
	Scheduled at convenient times (supported by a care coordinator or use of online booking system)	Reduces time/burden on patients/carers associated with attending uncoordinated appointments and coordinating care	
	A range of services and professionals can be accessed in one visit	Improves access to care (e.g. care is more timely) Reduces time/burden on patients/carers associated with attending uncoordinated appointments	
Stakeholders communicate effectively	Multi-disciplinary teams	Improves access to care (e.g. medical and non-medical aspects of care are considered) Reduces time/burden on patients/carers associated with attending uncoordinated appointments and coordinating care	*They normally ring me about a month before the appointment, 'Here's the list of everybody we think is currently involved with [son’s name], is this right?' which is really useful…We have a meeting for about an hour or so… The paediatrician is normally the person that leads the meeting… then she'll make some recommendations in terms of what she wants to see happen next… They are actually quite handy, those meetings, just to make sure that everybody knows what's going on. [carer, undiagnosed]*
	Patients have written care plans (including plans for acute episodes)	Improves access to care (e.g. facilitating proactive approach to care) Reduces time/burden on patients/carers associated with coordinating care	
	Patients, carers and professionals have a point of contact for specialist advice/liaison	Improves access to care (e.g. patients could access specialist information to guide self-care decisions when necessary)	*[respondent’s daughter]’s got her metabolic disorder guidelines, so when we go to A&E, we've kind of got a triage pass to get into triage straight away so that we don’t have to be hanging around waiting. [carer , diagnosed]*
	Technology is used to improve communication (including between specialists and local providers)	Improves access to care (e.g. local providers such as GPs and staff in Emergency Departments could access specialist information to guide care decisions when necessary) Reduces time/burden on patients/carers associated with coordinating care	

#### Having the support of a professional coordinator

One participant reported having a dedicated care coordinator within their specialist centre—they were a specialist nurse who acted as a point of contact for the patient, managed appointment scheduling and facilitated information sharing between relevant professionals. The benefits reported by the participant included having a bank of expert knowledge that they could always refer to and reducing the burden on their parent.

Participants suggested a professional coordinator could support patients and carers in a range of other ways including: coordinating care across different providers (including local ones), facilitating proactive care, and helping families access wider support services (such as funding opportunities (e.g. for specialist equipment within homes), social care and/or signposting to local charities who were seen as an important source of knowledge and support). Such support is likely to both improve access to care (especially locally, and both medical and non-medical) and reduce the time/burden on patients and carers associated with coordinating and fighting to access care.

Although it was agreed that having the support of a professional coordinator would be beneficial, there were diverse views about which professionals should fulfil the role of care coordinator, what type of training or background would be required, and when the support should be available. Therefore, different approaches may be required in different circumstances and/or with different patients. For example, some argued that a professional coordinator should be someone with a medical background or even someone who specialises in the condition. Whereas some others argued it should be someone with good organisation skills (not necessarily with a medical background). Some interviewees felt that care coordinators should only take on specific coordinating tasks as required, rather than having continuous, regular involvement. The support of a care coordinator may have greater benefits at key points in the patient journey—for example, post diagnosis, after hospital discharge and other transition periods (when care needs are being identified and treatment/support is being established) or during acute periods (to assist communication between local and specialist services).

Similarly, some patients and carers may prefer to retain more control over coordinating their care and preferences may change as personal circumstances do. For example, as parents return to work after maternity leave their capacity to be involved in coordinating tasks/attending appointments may decrease. Therefore, they may require the support of a coordinator more during this time.

*At the moment I'm on maternity leave so I have more time to do these things, but when I'm back at work, trying to organise everything and keep track of everything, time to book appointments and stuff is tricky. [carer, undiagnosed]*

#### Changing the organisation of appointments and clinics

As demonstrated by the experience of one patient, who was able to attend several appointments at the same location on the same day, the way that clinics are scheduled can reduce the time/burden on patients and carers, particularly the time/burden associated with travelling to and attending uncoordinated appointments. Other approaches were suggested. A family-centred approach to clinics (where more than one family member, with the same condition, could be seen on the same day) could improve communication and decision making between paediatric and adult services whilst reducing the costs to families associated with attending two sets of appointments.

Some approaches may not be feasible locally and although many participants recognised the value of receiving care at a specialist centre and felt it outweighed costs of travelling, the advantages of accessing specialist care locally or remotely were addressed. For example, the provision of services locally (such as via special schools or Child Development Centres) have the benefit of saving time on travel (and the associated travel costs) and being a familiar environment for the family. Many participants also realised the benefits of having some consultations virtually—particularly with specialists who were based far away and where face to face contact was not essential for every appointment. Interviewees commented on the usefulness of virtual appointment options—especially for those who experience fatigue.

Preferences regarding the timing and scheduling of appointments may also change depending on the needs of individual patients. For example, older children may be more able to cope with a full day of appointments, compared to a younger child.

*As he gets older I think sometimes it would be nice if we could have multiple appointments on the same day. That would really help, because you're not then having three appointments… When they're very small it's difficult and one appointment a day is better because attention spans are limited and everything else. [carer, undiagnosed]*

In summary, where the care is provided, what range of professionals and services are available at appointments, and how appointments are scheduled (timing, organisation) are all likely to influence how appointments impact on patients and carers. Interviewees felt that appointments should be scheduled to meet individual needs—one approach may not fit all.

#### Improving communication between stakeholders

One parent described an initiative (‘Team Around the Child’ meetings) which brought together all professionals involved in their child’s care (including both health and non-health professionals, local and specialist professionals). They argued that it helped promote proactive care and information sharing. Interviewees reported other ways that professionals can or could work as a multi-disciplinary team (MDT) including holding MDT clinics.

The need for a care plan (e.g. Education, Health and Care Plan (EHCP)) as a communication and coordination tool was evident in participants’ calls for everyone to be ‘singing off the same hymn sheet’, to agree an approach and to provide planned, rather than reactive, care. Similarly, guidelines or a care plan for acute scenarios were viewed as useful so that patients receive timely access to care, particularly locally.

Interviewees felt that having a point of contact for specialist advice would help with communication—for the family when making a decision about whether or not they need to go to hospital and/or for  professionals, such as local emergency staff, who require specialist information about the condition.

Interviewees highlighted ways in which they thought technology could improve communication and information sharing. This might be, for example, a computer system that allows relevant information to be accessed by all professionals involved in a patient's care as well as the patient/carer.

*I would like there to be one central website where I could log in… I could see when he’s due to see them next, I could message Consultants… and I could book appointments… maybe I could see his test results and stuff too… to actually access these digitally and online would be fantastic. [carer, undiagnosed]*

## Discussion

The study identified two key consequences of uncoordinated care on the patient and carer experience—delays and barriers in accessing care and additional time/burden. A range of impacts on patients and parents/carers were identified and grouped into three overarching themes relating to physical health, psychosocial impacts and financial impacts. Our findings also suggest ways in which service users felt negative impacts might be reduced, for example, with the support of a professional coordinator, using MDTs, care plans, technology and/or a point of contact to improve communication, and organising appointments to meet the needs of patients and families (by providing them locally or virtually where possible, offering a range of services in one visit, and scheduling them at a convenient time for the family). These findings are particularly valuable as they suggest the various ways in which services can be (re)designed to meet the needs of rare disease patients.

Our findings demonstrated that different levels of care coordination (e.g. the involvement of a care coordinator and how clinics/appointments are delivered) may be needed at different stages of the patient’s journey and/or to meet individual patient preferences. This highlights the need for coordination to take into account when new services or support are required (e.g. post diagnosis, transition to adult services) and when care is needed locally in non-specialist settings. There may also be key individual differences which affect the extent to which one might experience the consequences and impacts described in this paper. Some impacts may be magnified for some. For example, coordinating care may be more burdensome for a single parent without a wider support network. Such differences also need to be taken into account when considering how care should be coordinated.

Our data support previous findings from others on the burden of living with a rare and/or undiagnosed condition [10, 11] including: struggling to access appropriate expertise, support and information, financial strain (e.g. due to changes in employment) and psychosocial impacts (such as disruption to everyday life, living with uncertainty, and stress). However, this study offers a unique contribution to the existing knowledge base. It has identified the specific costs and potential benefits associated with how care is coordinated, rather than the more general consequences and needs associated with living with or caring for someone with a rare condition.

We did not quantify the impacts or costs to patients such as the extent of travel costs, but it could be argued that the impact of poor coordination on patients and families affected by rare conditions is likely to be greater than for those with common conditions because of several confounding factors. For example, many of the impacts of poor care coordination can be attributed to the time and financial commitments of attending appointments, such as cost of travel, loss of earnings and disruption to employment/education. These are likely to be substantial when one considers that rare disease patients visit many different professionals and services in short periods of time [4, 12] and specialist care may not be provided locally. Previous research shows that patients come up against a number of challenges within the healthcare system due to the rarity and complexity of their condition including: misdiagnoses and delays in diagnosis, a lack of information and support, and low availability of treatments [19]. This study suggests that uncoordinated care also contributes to the challenges faced by patients with rare diseases, particularly around accessing care and the burden of managing the condition.

Whilst the paper presents the impacts across three overarching themes (physical health, psychosocial and financial), it is important to note the interrelations between them. This is supported by previous research which found that parents faced financial challenges as a result of their caring role, which in turn was a major source of stress [11] and the findings of a recent study which highlighted a number of factors affecting the mental health of rare disease patients and their carers including trying to access services and support (including financial and non-medical support) [20]. Whilst it is helpful to categorise different impacts (for example, for identifying measures for evaluation and further research), the complex inter-dependencies found in reality should be recognised.

Findings support recent research which has found that patients and carers take on significant tasks in relation to care coordination and management [10, 21]. Having professional support to coordinate care could reduce the negative impact on patients and carers (and others), however, this support could take many different forms and there is further research and consultation required to establish the extent to which responsibility should be transferred from patient to professional.

Previous research has shown common challenges and needs across rare diseases, which are nevertheless unique to those with rare conditions [11, 19, 22]. Therefore, the collective experience of those with rare diseases is useful and should inform how care is coordinated in services for patients with rare conditions, including condition specific services. It is however also important to recognise the diversity of views among individuals and this study concludes that care coordination approaches should be flexible to meet the needs of different patients and carers at different time points.

Fifteen participants took part in this exploratory qualitative study. Whilst efforts were made to include a variety of individuals and experiences, the sample is not representative of all those affected by rare diseases. As an exploratory and qualitative study, the intention was not to recruit a representative sample of the rare disease population, but rather to focus on in-depth individual accounts which could support the development of data collection tools and interpretation of quantitative data in the CONCORD study. The data can be used to provide an insight into an under-researched area and to inform future research. In addition, although positive examples of care coordination were shared, it was challenges and gaps in care coordination that were more commonly felt amongst participants. Therefore, many of the approaches outlined in section three of the findings are based upon suggestions from participants rather than real life scenarios.

There may be other factors, not included here, which have an impact on patient and carers experience of care coordination. There may be institutional barriers such as: the availability of resources, structural barriers (such as the limitations of information systems within NHS organisations), and/or cultural obstacles to change. These factors should also be considered in the proposal and evaluation of new models of coordinated care.

This study identified how care could be coordinated in a way which benefits patients with rare conditions, and their carers. Further work is required to develop models of care coordination, assess their feasibility and evaluate them in practice to determine whether the potential benefits we have identified can be realised.

Our findings demonstrate the need for future research to collect the views of larger numbers of patients (and carers) and consider what might influence differences in preferences. This should also be extended to gather the views of professionals—those delivering care and supporting patients. This would give an insight into other possible impacts associated with how care is coordinated, including the impact on the NHS.

The CONCORD study will gather the views of larger numbers of people, and of health care professionals and patients and carers who are not necessarily members of a support group. This exploratory study has informed the development of an online survey, incorporating a Discrete Choice Experiment to evaluate preferences for different aspects of coordinated care. The findings will also inform the early phase of the development of a taxonomy which will provide a comprehensive description of the elements of care coordination and how they can be incorporated into models of service delivery. The taxonomy will form the basis of future cost analyses of different models of care.

## Conclusion

Our findings provide further evidence of both the challenges and the importance of coordinating care in the context of rare conditions. The findings presented in this exploratory qualitative study offer an in-depth view of how the patients and carers that we spoke to experience impacts of care coordination. The study identifies a range of negative consequences associated with poorly coordinated care including delays and barriers to accessing care, and additional time and burden on patients and carers, resulting in physical, psychosocial and financial impacts. In addition it proposes a number of ways that negative impacts might be reduced for patients and carers including: having the support of a care coordinator, having clinics and appointments organised in a way which better meet patient needs, and effective communication between professionals and services. The findings stress the importance of approaches to care coordination which are flexible to individual needs and fit for purpose throughout the patient journey. Whilst the findings provide an in-depth view of a small number of participant’s experiences, they may not generalise to all patients and carers living with rare conditions. The research should influence future service developments (and the evaluation of such developments).

## Supplementary Information

**Additional file 1.** Interview guide for patients and carers.

## Data Availability

Data supporting the conclusions in this article are included within the article itself. Further data is available from the corresponding author on request.

## Abbreviations

UK: United Kingdom
NHS: National Health Service
CONCORD: COordiNated Care Of Rare Diseases
SWAN UK: Syndromes Without A Name UK
IC: Iterative categorisation
GP: General practitioner
MDT: Multi-disciplinary team
EHCP: Education, Health and Care Plan

## Footnotes

1 One participant had a child who had been diagnosed with the same condition as them – the participant is recorded as a patient (rather than parent) in the Table 1, although they were able to talk from both perspectives.

2 It is important to note that participants are likely to have interpreted ‘specialist centre’ differently as no definition was provided. Further detail was captured during the interviews and is described in the results section.
