# Supplementary material for: How are patients with rare diseases and their carers in the UK impacted by the way care is coordinated? An exploratory qualitative interview study
Source: Orphanet J Rare Dis. 2021 Feb 10;16:76. doi: 10.1186/s13023-020-01664-6 (PMC7874609; doi:10.1186/s13023-020-01664-6)
Supplement: Supplementary file 1 — Additional file 1. Interview guide for patients and carers. [file 13023_2020_1664_MOESM1_ESM.docx]

**UCL DEPARTMENT OF APPLIED HEALTH**

**RESEARCH**

**INTERVIEW GUIDE FOR PATIENTS AND CARERS**[1]

**Title of Study:**

COordiNated Care Of Rare Diseases (CONCORD)

**Purpose of interviews:**

- To understand the burdens associated with living with and managing rare and undiagnosed conditions
- To get a better understanding for how services are coordinated for patients with rare and undiagnosed conditions (and how patients and families experience these different models of care)
- To identify the important elements of care coordination and relevant cost components (financial and non-financial)
- To inform development of survey/DCE and taxonomy

**Interview questions:**

***About you and your care***

Please could you tell me a bit about your condition?

Please could you tell me about the ongoing care[2] you require?

Please could you tell me about how your care is currently organised? *(Possible prompts: Who provides your care? When I ask you who coordinates your care who do you think of? How often do you see them, and where? How do they communicate with you/each other?)*

How satisfied are you with how your care is currently organised? Please explain your answer. *(Prompt participant to rank their satisfaction on a scale - very satisfied/mostly satisfied/neither/mostly unsatisfied/very unsatisfied).*

What would the ideal service for you look like? Please could you tell me why this would be ideal?

***Defining care coordination***

What does ‘care coordination’ mean to you?

What are the most important elements of care coordination for you? Please could you tell me a bit about why these are important? [3]

Are the importance of these elements likely to change? (e.g. over time) If so, why? If not, why not?

***Costs and benefits associated with the coordination of care[4]***

Have there been any **benefits** to you in relation to your care that you think have resulted from the way your care is coordinated? If yes, what are they?

Have these benefits had an impact on you/your family?

If yes, can you tell me how?

If not, can you tell me why?

Have these benefits affected your ability to access care?

If yes, can you tell me how?

If not, can you tell me why?

Have there been any **costs** to you in relation to your care that you think have resulted from the way your care is coordinated? If yes, what are they?

Have these costs had an impact on you/your family?

If yes, can you tell me how?

If not, can you tell me why?

Have these costs affect your ability to access care?

If yes, can you tell me how?

If not, can you tell me why?

Have there been any **financial costs or benefits** to you in relation to your care that you think have resulted from the way your care is coordinated? If yes, what are they?

Have these had an impact on you/your family?

If yes, can you tell me how?

If not, can you tell me why?

Do these affect your ability to access care?

If yes, can you tell me how?

If not, can you tell me why?

Is there anything else you would like to say?

*Notes for researchers:*

*[1] The questions will be adapted for patients/parents/carers (e.g. tell me about your child’s condition)*

*[2] Interviewees will be asked about all aspects of their care, but will be prompted to focus on health care. For those who may have recently transitioned from paediatric care - ask about the care they required at this time.*

*[3] Prompts will be developed from emerging findings of scoping review.*

*[4] The interviewer will encourage participants to think about different types of costs and benefits (including impact on time, psychological/emotional impact) – for them and others*
